# Levetiracetam-Induced Rhabdomyolysis Reversed by Discontinuation: A Case Report

**DOI:** 10.7759/cureus.48955

**Published:** 2023-11-17

**Authors:** Jacob S Kazmi, Nabila Albarghouthy, Randy Ramsaywak

**Affiliations:** 1 Department of Neurology, Donald and Barbara Zucker School of Medicine at Hofstra/Northwell, New York, USA; 2 Department of Neurology, Montefiore Medical Center, Wakefield Campus, New York, USA; 3 Department of Medicine, Montefiore Medical Center, Wakefield Campus, New York, USA

**Keywords:** drug-related side effects and adverse reactions, case report, creatine kinase, levetiracetam, rhabdomyolysis

## Abstract

Rhabdomyolysis has been reported as a rare side effect of levetiracetam, a first-line anti-epileptic medication. We report the case of a 64-year-old man who presented to the medical center after suffering an unwitnessed seizure. Following the initiation of levetiracetam, the patient’s serum creatine kinase (CPK) levels rose rapidly and remained elevated for multiple days. However, the patient did not report any symptoms of acute rhabdomyolysis. Following discontinuation of the medication CPK levels normalized, suggesting that this is a reversible adverse effect of levetiracetam. The patient made a complete recovery and did not display any seizure activity after the initial presentation. This seemingly more common side effect could cause further damage, particularly to the kidneys, and should be monitored closely by prescribing clinicians.

## Introduction

Levetiracetam is a commonly used, second-generation anti-epileptic medication that is thought to elicit anti-seizure effects through inhibition of synaptic vesicle glycoprotein 2A receptor and voltage-gated calcium channel blockade [1,2]. Levetiracetam is generally well-tolerated, with known adverse effects of asthenia, drowsiness, fatigue, headache, and infection [3]. Additional behavioral adverse events such as irritability, anger, and aggression have been reported [4]. The complication of rhabdomyolysis or other skeletal muscle injury after levetiracetam use has been rarely reported.

Drug-induced rhabdomyolysis has been associated with a number of seemingly unrelated drug classes, including 3-hydroxy-3-methyl-glutaryl-coenzyme A (HMG-CoA) reductase inhibitors, macrolide antibiotics, tyrosine kinase inhibitors, and benzodiazepines [5]. While clear mechanisms have not been established, mitochondrial dysfunction and dysregulation of calcium homeostasis seem to be common threads in cases of drug-induced rhabdomyolysis [6].

Here we describe a patient who exhibited an elevated serum creatine kinase (CPK) level in the absence of rhabdomyolysis symptoms after treatment with levetiracetam, which was alleviated by discontinuation of therapy.

## Case presentation

**Figure 1:**
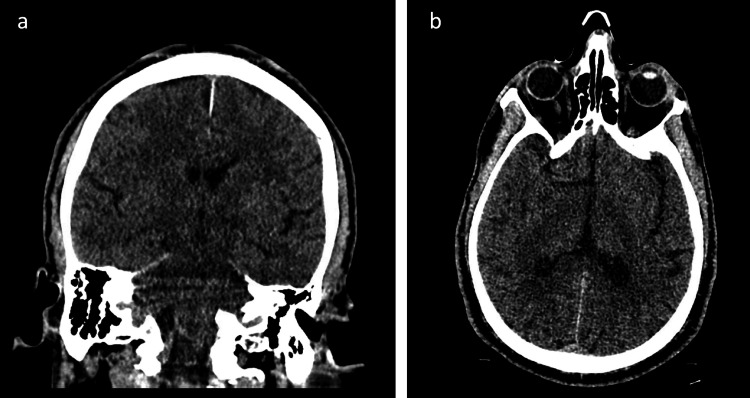
Coronal (a) and axial (b) non-contrast computed tomography (CT) images taken at arrival to the emergency department

A 64-year-old man with a previous medical history of hypertension, hyperlipidemia, generalized anxiety, prostate cancer, and an unknown seizure disorder was found unconscious, foaming around the mouth, and incontinent of urine at home. A paramedic team administered a valium dose of 10 mg intravenously before arrival at the emergency department. While in the emergency department, he was noted to be extremely agitated and uncooperative, prompting sedation with a combination of lorazepam and midazolam. As per the neurology team, the patient was loaded with 4,500 mg intravenous levetiracetam for seizure prevention before transfer out of the emergency department. Computed tomography imaging performed at the time was remarkable for an acute bilateral subdural hematoma along the tentorium, which was determined by neurology and radiology teams to be a result of trauma sustained during the seizure episode (Figure 1). CPK level upon presentation to the emergency department was slightly elevated at 660 U/L, which was attributed to muscular injury during the seizure episode. The altered mental status in combination with pyrexia raised clinical suspicion of infection involving the central nervous system, and the patient was treated with intravenous vancomycin and dexamethasone before admission to the medical intensive care unit for monitoring.

On day two of admission, lumbar puncture was performed and returned unremarkable for viral encephalitis or meningitis. The patient was diagnosed with rhabdomyolysis on day two, given a serum CPK level of 19,841 U/L in the setting of decreased urine output and continued altered mental status. The patient denied symptoms of myalgia, weakness, or swelling. Aggressive rehydration with intravenous fluids was initiated by the medical team and intravenous administration of levetiracetam was continued at 1 g every 12 hours. Neurologically, the patient’s seizure symptoms resolved, but a general amnesic syndrome and confusion persisted. Despite attempts to control rhabdomyolysis, the patient’s CPK level remained elevated through day five of admission (17,472 U/L), and various etiologies of rhabdomyolysis were excluded, including muscular trauma, IV drug abuse, alcoholism, acute infection, and electrolyte dysregulation.

**Figure 2:**
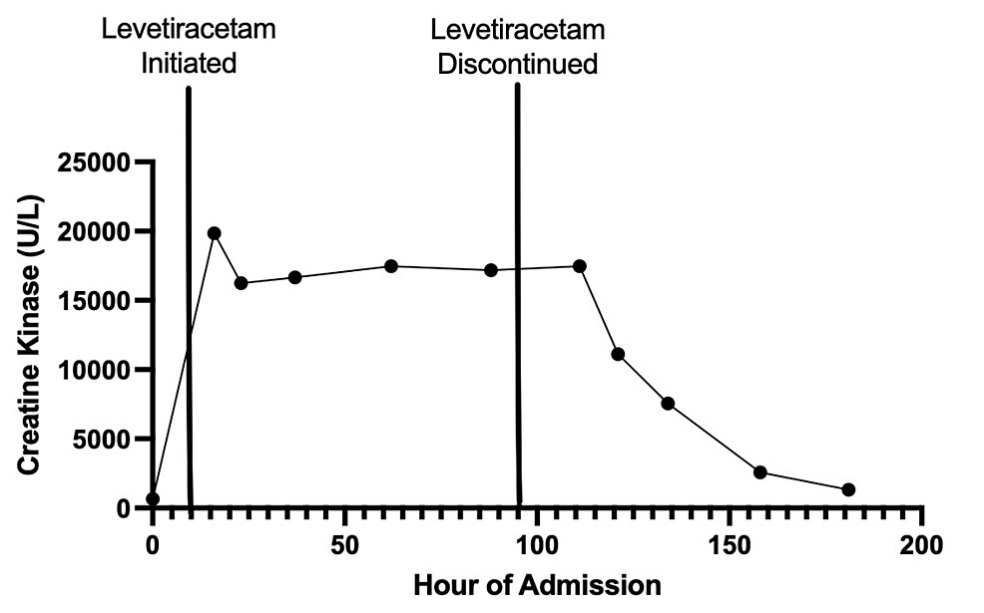
Serum creatine kinase levels over the course of admission

By day five, the patient’s rising CPK level despite continued hydration raised the suspicion of levetiracetam-induced rhabdomyolysis as previously reported in the literature. The neurology team recommended switching the patient’s anti-epileptic regimen to 50 mg of oral lacosamide every 12 hours. By the morning of day six, the patient’s CPK level was still elevated at 17,479 U/L but began to trend downwards to 2,565 U/L the morning of discharge on day eight (Figure 2). The patient’s seizure symptoms were completely resolved by discharge; however, continued confusion was noted and was suspected to be either a temporal lobe pathology or acute worsening of a previously undiagnosed cognitive deficit.

## Discussion

Levetiracetam is a well-tolerated and commonly used anti-epileptic medication with a mild side effect profile; however, the rare adverse event of rhabdomyolysis has been previously reported [7-10]. A review of the United States Food and Drug Administration Adverse Event Reporting System Database in late 2023 revealed 960 instances of rhabdomyolysis or elevated serum CPK after levetiracetam use since 2016 [11]. Given the close temporal association between levetiracetam initiation and CPK elevation, a diagnosis of drug-induced rhabdomyolysis is highly likely. To our knowledge, this is the eldest patient reported to have this adverse effect of levetiracetam.

The patient did not report symptoms of rhabdomyolysis throughout the course of hospitalization or post-discharge, which has also been reported [10]. The probable diagnosis is strengthened by the fact that discontinuation of levetiracetam and initiation of lacosamide was followed by a drop in serum CK levels. This is in line with previous reports of levetiracetam-induced rhabdomyolysis and suggests a reversible mechanism of injury.

As with most drug-induced rhabdomyolysis cases, the precise mechanism of levetiracetam-induced rhabdomyolysis remains unclear. One identified potential mechanism is the potentiation of cholinergic signaling at neuromuscular junctions, which may over-excite muscles leading to their injury [12]. We additionally propose the possibility of an underlying channelopathy, which when challenged with levetiracetam may lead to a state of hyperexcitability or hypermetabolism within skeletal muscle.

## Conclusions

In conclusion, we believe that this 64-year-old man exhibited the rare adverse event of asymptomatic rhabdomyolysis after leviteracetam administration. The lack of muscular symptoms may delay the diagnosis of levetiracetam-induced rhabdomyolysis patients. Additionally, seizure episodes may be followed by transient rises in serum CK levels as a result of prolonged contraction, which may cloud the clinical picture. The collection of blood samples prior to treatment initiation can help to identify seizure-induced rhabdomyolysis. While more studies are required to understand which patients are at risk for muscular injury in the setting of levetiracetam therapy, clinicians should be aware of this seemingly more common adverse effect.
